# Supplementary material for: From sequence to enzyme mechanism using multi-label machine learning
Source: BMC Bioinformatics. 2014 May 19;15:150. doi: 10.1186/1471-2105-15-150 (PMC4229970; doi:10.1186/1471-2105-15-150)
Supplement: Additional file 2 — Java code of ml2db. Additional file ml2db_code.tar.gz contains the Java source code to run the multi-label machine learning experiments and save the results to database. The code’s Javadoc is included. [file 1471-2105-15-150-S2.zip › additional file 2/ml2db/ml2db/doc/main/ml2db/mulan/predict/PredictionsExperimentTable.html]

PredictionsExperimentTable


JavaScript is disabled on your browser.


- Overview
- Package
- Class
- Use
- Tree
- Deprecated
- Index
- Help

- Prev Class
- Next Class

- Frames
- No Frames

- All Classes

- Summary:
- Nested |
- Field |
- Constr |
- Method

- Detail:
- Field |
- Constr |
- Method


main.ml2db.mulan.predict

## Class PredictionsExperimentTable

- java.lang.Object
- - uk.ac.standrews.utils.main.database.Table
  - - main.ml2db.mulan.predict.PredictionsExperimentTable

- ---

    

  ```
  public class PredictionsExperimentTable
  extends uk.ac.standrews.utils.main.database.Table
  ```

- - ### Field Summary

    Fields

    | Modifier and Type | Field and Description |
    | --- | --- |
    | `static java.lang.String` | `PREDICTION_EXPERIMENTS_TABLE_NAME` the name for the table to store the predictions experiments records |

    - ### Fields inherited from class uk.ac.standrews.utils.main.database.Table

      `m_writer`
  - ### Constructor Summary

    Constructors

    Constructor and Description

    `PredictionsExperimentTable(uk.ac.standrews.utils.main.database.DbManager dbManager)`
  - ### Method Summary

    Methods

    | Modifier and Type | Method and Description |
    | --- | --- |
    | `static java.util.Vector<uk.ac.standrews.utils.main.database.TableColumn>` | `getColumnDefinitions()` |
    | `int` | `saveRow(java.lang.String startTime, java.lang.String algorithm, int trainArffId, int testArffId, java.lang.String trainArffData, java.lang.String testArffData, int fold)` |

    - ### Methods inherited from class uk.ac.standrews.utils.main.database.Table

      `getColumnNames, getColumns, getDbManager, getTableCreator, getTableName, getTableReader, getTableRow, getTableWriter, hasKey, initialise, initialise, name, saveRow`
    - ### Methods inherited from class java.lang.Object

      `equals, getClass, hashCode, notify, notifyAll, toString, wait, wait, wait`

- - ### Field Detail


    - #### PREDICTION\_EXPERIMENTS\_TABLE\_NAME

      ```
      public static final java.lang.String PREDICTION_EXPERIMENTS_TABLE_NAME
      ```

      the name for the table to store the predictions experiments records

      See Also:
      :   Constant Field Values
  - ### Constructor Detail


    - #### PredictionsExperimentTable

      ```
      public PredictionsExperimentTable(uk.ac.standrews.utils.main.database.DbManager dbManager)
      ```
  - ### Method Detail


    - #### saveRow

      ```
      public int saveRow(java.lang.String startTime,
                java.lang.String algorithm,
                int trainArffId,
                int testArffId,
                java.lang.String trainArffData,
                java.lang.String testArffData,
                int fold)
      ```


    - #### getColumnDefinitions

      ```
      public static java.util.Vector<uk.ac.standrews.utils.main.database.TableColumn> getColumnDefinitions()
      ```


- Overview
- Package
- Class
- Use
- Tree
- Deprecated
- Index
- Help

- Prev Class
- Next Class

- Frames
- No Frames

- All Classes

- Summary:
- Nested |
- Field |
- Constr |
- Method

- Detail:
- Field |
- Constr |
- Method
